# Determinants of baseline seroreactivity to human papillomavirus type 16 in the Ludwig-McGill cohort study

**DOI:** 10.1186/s12879-014-0578-0

**Published:** 2014-11-18

**Authors:** Patrícia S de Araujo-Souza, Agnihotram V Ramanakumar, João M G Candeias, Patrícia Thomann, Andrea Trevisan, Eduardo L Franco, Luisa L Villa, Ludwig-McGill Cohort Study

**Affiliations:** Ludwig Institute for Cancer Research, São Paulo, Brazil; Department of Immunobiology, Fluminense Federal University, Niterói, Brazil; Program of Cellular Biology, Brazilian National Cancer Institute, Rio de Janeiro, Brazil; Department of Oncology, McGill University, Montreal, Canada; Department of Microbiology and Immunology, Institute of Biosciences, Universidade Estadual Paulista, Botucatu, Brazil; Institute of Gynaecological Cancer Research, São Paulo, Brazil; Division of Gynecologic Oncology, CHUM - Université de Montréal, Montreal, Canada; Department of Epidemiology, Biostatistics, and Occupational Health, McGill University, Montreal, Canada; Molecular Biology Laboratory, São Paulo Cancer Institute, ICESP, São Paulo, Brazil; Department of Radiology and Oncology, School of Medicine, University of São Paulo, São Paulo, Brazil; School of Medicine, University of São Paulo and Santa Casa de São Paulo, São Paulo, Brazil

**Keywords:** Serology, HPV, Natural antibodies, HPV16 seropositivity

## Abstract

**Background:**

Immunity plays an important role in controlling human papillomavirus (HPV) infection and associated lesions. Unlike infections caused by other viruses, natural HPV infection does not always result in a protective antibody response. Therefore, HPV antibodies are also considered markers of cumulative exposure. The aim of this study was to identify determinants of HPV16 seroreactivity at enrollment among women from the Ludwig-McGill cohort, a natural history study of HPV infection and risk of cervical neoplasia.

**Methods:**

HPV16 serology was assessed by ELISA for L1 and L2 capsid antigens, while HPV typing and viral load measurements were performed by PCR-based methods. The associations were analyzed by unconditional logistic regression.

**Results:**

Of 2049 subjects, 425 (20.7%) were strongly seropositive for HPV16. In multivariate analysis, seroreactivity was positively correlated with age, lifetime number of sexual partners, frequency of sex, and HPV16 viral load, and negatively associated with duration of smoking.

**Conclusions:**

HPV16 seroreactivity is determined by factors that reflect viral exposure.

## Background

Persistent infection by human papillomavirus (HPV) is a necessary cause of cervical cancer, but only a small proportion of HPV positive women develop cervical lesions. HPV infection is considered the most common sexually transmitted infection worldwide. Most sexually active individuals are likely to be exposed to HPV infection at some point in their lives [1]. However, most infections seem to clear spontaneously within 12 to 24 months [2],[3] as a result of either humoral immune response or cell mediated mechanisms [4],[5].

The high frequency of HPV-related diseases in immunocompromised individuals underscores the important role of immune response to control HPV infection [6]. Indeed, HPV exhibits several mechanisms to avoid immune system recognition. HPV's productive life cycle is coupled to the cellular differentiation cycle of infected host cells. As such, the most immunogenic capsid proteins are only expressed in keratinocytes in the upper layers of the epithelium where the immune system has limited access. Moreover, the expression of early proteins is maintained at very low levels [7]. Since there is no viremia and no cell lysis upon viral shedding, the low availability of antigens and the absence of danger signals also contribute to keep HPV infection unknown to the host's immune system.

In contrast with other viral infections in which natural exposure results in a protective antibody response, only about half of the women infected with HPV have detectable levels of anti-HPV serum antibodies. Furthermore, about half of these HPV seropositive women produce neutralizing antibodies capable of preventing initial phases of infection [8]. Therefore, HPV antibodies generated against HPV natural infections are not necessarily protective [9],[5], but always work as markers of cumulative exposure. High antibody titers induced by HPV vaccines correlate with protection to HPV infections and lesions [10],[11].

The Ludwig-McGill cohort study is a longitudinal investigation of the natural history of HPV infection and related lesions of the uterine cervix. About 2,500 women were followed for up to 10 years. The aim of the present study was to identify determinants of HPV16 seroreactivity at enrollment among women from the Ludwig-McGill Cohort Study.

## Methods

### Subject recruitment

A detailed description of the design and methods for subject recruitment, scheduled returns, epidemiologic data, medical procedures, and biological sampling has been described elsewhere [12]. Briefly, 2528 women with permanent residence in the city of Sao Paulo, Brazil, were recruited between 1993 and 1997 and followed up until 2005. These women attended a comprehensive maternal and child health program for low-income families at a public hospital (Hospital e Maternidade Vila Nova Cachoeirinha). They were between 18 and 60 years old, had an intact uterus, no current referral for hysterectomy, and did not report treatment for cervical disease in the previous 6 months. Cervical cell specimens were collected for cytologic and HPV DNA analyses and blood samples were taken at each of four visits, 4-months apart, in the first year, followed by annual and semester visits thereafter for HPV16 serology and cervical sampling, respectively. Subjects answered a nurse-administered questionnaire to collect information on sociodemographics, lifestyle, and sexual, reproductive, and contraceptive characteristics. Subjects gave a signed informed consent. This study was approved by the Ludwig Institute for Cancer Research São Paulo Branch IRB and by the IRB of the Hospital e Maternidade Escola Vila Nova Cachoerinha Dr Mario Altenfender, São Paulo, Brazil.

### HPV Serology

Serum samples were separated from clotted blood specimens and stored at -20°C until testing. An ELISA technique was used for semi-quantitative measurement of IgG antibodies to HPV16. Recombinant HPV16 virus-like particles, constructed with L1 capsid protein, were prepared in the baculovirus system. An initial batch was kindly donated by Dr. John Schiller, Laboratory of Cellular Oncology, U.S. NIH and subsequent batches were kindly donated by Dr. Kathryn Jansen, Merck Laboratories, USA. The ELISA protocol was performed as described elsewhere [13],[14]. Briefly, polystyrene ELISA microtiter plates were coated with 50 μL aliquots of a solution of 2 mg of HPV16 virus-like-particles per 100 mL of PBS and incubated for 1.5 hours at 37°C. Plates were washed three times with calcium- and magnesium-free PBS and then incubated with serum samples diluted 1:10 and 1:50 in PBS containing 0.5% skim milk and 0.1% newborn calf serum (PBS-MNCS) for 2.5 hours at 37°C. Following repeated washings, plates were incubated for 1 hour at room temperature with 50 μL aliquots of a conveniently diluted (by prior block titration) peroxidase-labeled anti-IgG conjugate. Following an additional washing cycle, a chromogen substrate mixture (0.1 mg/mL O-phenylenediamine and 0.003% hydrogen peroxide diluted in 0.15 mol/L PBS; pH 6.0) was added to the wells. Absorbances were read at 490 nm in a colorimetric plate reader after 45 minutes. Replicate blank wells, with PBS-MNCS instead of diluted serum samples, and a control human serum pool were included in all plates. The latter was included to control the inter- and intra-assay variation in reactivity that is inherent to immunoenzymatic techniques. A single batch of this serum pool (aliquoted and kept frozen at -20°C) was prepared beforehand from dozens of blood bank and normal clinical laboratory specimens from female adult donors at the AC Camargo Hospital in Sao Paulo and maintained at the Sao Paulo Branch of the Ludwig Institute for Cancer Research. An aliquot from this pool was thawed and processed in the same manner as all study serum samples included in each ELISA run and the same serum pool was used throughout the study. Absorbances were corrected for the fluctuation in seroreactivity of this serum pool as previously described [14].

Seroreactivity was expressed as normalized absorbance ratios (NAR) by dividing the mean blank-subtracted (net) optical densities (ODs) by the equivalent values of the control serum pool included in the same plate in triplicate, using different dilutions. An average NAR (AvgNAR) for each serum sample was then calculated based on two dilutions: AvgNAR = (NAR10+ NAR50)/2, where NAR10 and NAR50 are the results with the 1:10 and 1:50 dilutions, respectively. In a previous report we described the reproducibility of the assay and calculation of seroreactivity and demonstrated that this method is suitable to minimize measurement error in ELISA assays [14]. We arbitrarily defined a high level of seroreactivity as one in the upper quintile of the distribution of AvgNAR results.

Since our goal was to investigate the correlates of seroreactivity at baseline in the cohort, only the serum samples from the first two visits were considered. Of the 2462 eligible subjects at the enrollment visit, 2185 subjects were followed up at the second visit, 4 months later. Serological results were available for 1745 and 1656 women in the first and second visits, respectively. To maximize the availability of serological data for analysis we imputed to the woman the serological result from the second visit if the one for the enrollment visit was missing, which resulted in 304 imputations. Therefore, a total of 2049 subjects had baseline IgG seroreactivity anti-HPV16, under the assumption that in a 4-month period there would not be any important fluctuations in serological results. Questionnaire-based information was derived from the interviews conducted at the enrollment visit.

### HPV DNA detection and typing

DNA was extracted from ecto- and endocervical cell samples and assessed for DNA quality by amplification of a 268-bp β-globin gene fragment [15]. MY09/11 and PGMY PCR protocols were used for HPV detection [16],[17], the former initially for the first few visits and then replaced by the latter in subsequent visit in the cohort. Each PCR reaction included negative and positive controls. HPV typing was performed by hybridization with individual oligonucleotide probes and ambiguous results were resolved by using restriction fragment length polymorphism analysis of the L1-amplified fragment with a set of restriction enzymes. The genotypes tested included high oncogenic risk (HR-) HPVs 16, 18, 31, 33, 35, 39, 45, 51, 52, 56, 58, 59, 66, 68, 73, and 82, and low oncogenic risk (LR-) HPV types 6, 11, 26, 32, 34, 40, 42, 44, 53, 54, 57, 61, 62, 67, 69, 70, 71, 72, 81, 83, 84, and 89 (unknown types considered LR-HPVs) [18],[19]. Specimens were tested blindly with respect to all other subject-specific results and precautions were taken to prevent contamination. Samples that were negative for both HPV and β-globin were considered inadequate for analysis. Only the HPV genotype results at the first visit were considered in the current analysis.

### Viral load

Quantification of viral load in HPV-positive samples was performed by low-stringency PCR with HPV generic GP5/6 primers [20],[21]. DNA from cervical cell lines Caski and HeLa were included in every assay as positive controls. Standards were also included to enable the quantification of viral load, and consisted of mixtures containing varying amounts of HPV16 reference plasmid added to a constant background of normal human DNA (corresponding to 4, 20, 100, 500, and 2,500 viral copies per cell, assuming a diploid genome). In low stringency conditions these primers amplify a specific L1 region in the HPV genome and a human genome sequence. The signal strength of the two silver-stained gel bands was quantified by densitometry. The logarithm of the ratio between these two bands is directly proportional to the logarithm of the amount of HPV DNA in individual samples; linear interpolation in a standard curve constructed with the results from control mixtures allows proper quantification of viral load expressed in viral copies per cell harboring a diploid genome.

### Statistical analysis

The associations between HPV16 serological reactivity and socioeconomic determinants, lifestyle factors, reproductive health factors, or clinical and viral characteristics were measured by unconditional logistic regression. We calculated odds ratios (OR) and respective 95% confidence intervals (CI) to gauge the magnitude and precision of the associations. Age and HPV16 DNA positivity were considered potential confounders or mediators and were thus included as covariates to be adjusted for in all exploratory models. Age was considered as 4 categories (18-24, 25-34, 35-44, and 45+ years). We also sought to identify a subset of independent correlates of anti-HPV16 seroreactivity via a stepwise multivariate logistic regression strategy with backwards elimination of variables (p = 0.15 for elimination). We used STATA statistical software (version 10.0) in all analyses.

## Results

To analyze the predictors of serological response in the cohort, we arbitrary selected the top 20% of seroreactivity results of 2049 women who had HPV16 serological results. Consequently we identified 425 women for the purpose of the current analysis.

Table 1 shows the associations between HPV16 seroreactivity and sociodemographic variables. Seroreactivity increased significantly with age, and decreased with higher levels of education and income, albeit non-significantly. These associations were not attenuated upon adjustment for cervical HPV16 DNA positivity.

**Table 1:** **Associations between sociodemographic variables and serological reactivity for HPV16**

Characteristic and categories	HPV16 seroreactivity 425/1624 (%)*	OR (95%CI)**	
		Unadjusted	Adjusted (HPV16 and age)
**Age:**			
<25	64/344 (15.7)	1.0	1.0
25-34	161/646 (20.0)	1.33 (0.97-1.84)	1.38 (0.97-1.84)
35-44	136/471 (22.4)	1.55 (1.11-2.15)	1.60 (1.15-2.23)
45+	64/163 (28.2)	2.11 (1.42-3.12)	2.16 (1.45-3.22)
**Ethnicity:**			
White	226/900 (20.1)	1.0	1.0
Non-white	139/479 (22.5)	0.87 (0.69-1.08)	0.84 (0.68-1.06)
**Marital Status:**			
Single	49/162 (23.2)	1.0	1.0
Married/With partner	330/1339 (19.8)	0.81 (0.58-1.14)	0.76 (0.58-1.14)
Widowed/Separated	46/122 (27.4)	1.24 (0.78-1.98)	1.01 (0.54-1.09)
**Education:**			
<Elementary	114/345 (24.8)	1.0	1.0
Elementary	240/964 (19.9)	0.75 (0.58-0.97)	0.81 (0.62-1.05)
High School	63/262 (19.4)	0.72 (0.51-1.02)	0.85 (0.59-1.21)
College/University	8/51 (13.6)	0.47 (0.21-1.03)	0.51 (0.23-1.12)
**Income Quartiles**:			
1^st^ Quartile	112/359 (23.8)	1.0	1.0
2^nd^ Quartile	114/431 (20.9)	0.84 (0.63-1.13)	0.88 (0.65-1.18)
3^rd^ Quartile	92/406 (18.5)	0.73 (0.53-0.98)	0.71 (0.52-0.97)
4^th^ Quartile	94/399 (19.1)	0.75 (0.55-1.02)	0.74 (0.54-0.99)

*Frequencies above and below the threshold for the top 20% quintile and percentage with high seroreactivity; **Odds ratios and respective 95% confidence intervals. First category in each variable is the referent.

Table 2 shows the associations with lifestyle and sexual risk factors. Current smoking is associated with a moderate decrease in likelihood of being strongly seroreactive for HPV16. As expected, seroreactivity increases when risk factors that reflect HPV exposure are considered, particularly those that indicate long-term, cumulative effects, such as lifetime number of partners and age at first intercourse. Frequency of sex but not recent partners was associated with an increase in strong seroreactivity, also in a dose-response manner as markers of cumulative exposure. Practice of oral or anal sex was not related to seroreactivity. All significant associations persisted after controlling for age and HPV16 DNA positivity.

**Table 2:** **Associations between lifestyle factors and serological reactivity for HPV16**

Characteristic and categories	HPV16 seroreactivity 425/1624 (%)*	OR (95%CI)**	
		Unadjusted	Adjusted (HPV16 and age)
**Smoking:**			
Never	216/768 (22.0)	1.0	1.0
Current	124/581 (17.6)	0.75 (0.59-0.97)	0.74 (0.58-0.98)
Former	85/275 (23.6)	1.09 (0.82-1.46)	1.06 (0.79-1.42)
**Smoking duration:**			
Never	216/768 (22.0)	1.0	1.0
<=10 years	86/379 (18.5)	0.81 (0.61-1.07)	0.81 (0.61-1.07)
11+ years	95/413 (18.7)	0.82 (0.62-1.07)	0.62 (0.57-0.98)
**Drinking:**			
Never	131/556 (19.1)	1.0	1.0
Ever drinker	291/1064 (21.5)	1.16 (0.92-1.47)	1.16 (0.92-1.47)
**Lifetime sexual partners:**			
0-1	132/770 (14.6)	1.0	1.0
2-3	162/555 (22.6)	1.70 (1.32-2.19)	1.70 (1.32-2.20)
4-5	84/181 (31.7)	2.71 (1.97-3.72)	2.56 (1.97-3.53)
6+	47/117 (28.7)	2.34 (1.59-3.44)	2.29 (1.55-3.37)
**Sexual partners, last 5 years:**			
0-1	321/1262 (20.3)	1.0	1.0
2-3	83/290 (22.2)	1.12 (0.86-1.48)	1.17 (0.88-1.55)
4+	20/71 (22.0)	1.10 (0.66-1.84)	1.30 (0.77-2.19)
**Sexual partners, last year:**			
0-1	400/1534 (20.7)	1.0	1.0
2-3	15/69 (17.9)	0.83 (0.47-1.47)	0.90 (0.51-1.60)
4+	2/5 (28.6)	1.53 (0.29-7.93)	1.89 (0.36-9.87)
**Age at first sexual intercourse**:			
20-50	89/446 (16.6)	1.0	1.0
18-19	89/344 (20.6)	1.30 (0.93-1.79)	1.52 (1.08-2.13)
16-17	107/417 (20.4)	1.29 (0.94-1.76)	1.60 (1.15-2.23)
<=15	140/417 (25.1)	1.68 (1.25-2.27)	2.18 (1.59-3.00)
**Frequency of sex (weekly):**			
0-1 times	224/865 (20.6)	1.0	1.0
2-3	133/576 (18.8)	0.89 (0.70-1.13)	0.95 (0.75-1.22)
4-5	42/123 (25.5)	1.31 (0.90-1.92)	1.53 (1.04-2.26)
6+	25/58 (30.1)	1.66 (1.01-2.72)	1.77 (1.07-2.92)
**Practice of anal sex:**			
Never	254/1018 (20.0)	1.0	1.0
Ever	171/606 (22.0)	1.13 (0.91-1.41)	1.14 (0.92-1.42)
**Practice of oral sex:**			
Never	195/739 (20.9)	1.0	1.0
Ever	230/885 (20.6)	0.98 (0.79-1.22)	1.08 (0.87-1.34)

*Frequencies above and below the threshold for the top 20% quintile and percentage with high seroreactivity; **Odds ratios and respective 95% confidence intervals. First category in each variable is the referent.

Results for reproductive health characteristics that could influence HPV seroreactivity are presented in Table 3. To analyze possible hormonal or artifactual influences from menstrual cycle, we classified women according to the time between onset of last menstrual period and date of specimen collection as follicular phase (before 14 days) or luteal phase (after 14 days). Parity was associated significantly with having a strong anti-HPV16 response in a dose-response manner. However, this effect was considerably attenuated and lost statistical significance upon adjustment for age and HPV DNA positivity. No other factors were significantly associated with seroreactivity, regardless of analytical framework.

**Table 3:** **Associations between reproductive health characteristics and serological reactivity for HPV16**

Characteristic and categories	HPV16 seroreactivity 425/1624 (%)*	OR (95%CI)**	
		Unadjusted	Adjusted (HPV16 and age)
**Oral Contraceptives**:			
Never	60/264 (18.5)	1.0	1.0
<6 years	235/885 (21.0)	1.17 (0.85-1.60)	1.21 (0.88-1.67)
6+ years	130/475 (21.5)	1.20 (0.86-1.69)	1.07 (0.76-1.51)
**Inter-uterine Device (IUD):**			
Never	281/1010 (21.8)	1.0	1.0
Ever	144/614 (19.0)	0.84 (0.67-1.05)	0.88 (0.70-1.10)
**Condom Use:**			
Never	177/600 (22.8)	1.0	1.0
Rarely	144/574 (20.1)	0.85 (0.66-1.09)	0.88 (0.66-1.08)
Sometimes/always	98/423 (18.8)	0.78 (0.59-1.02)	0.84 (0.63-1.12)
Menstrual Cycle***:			
Follicular phase	185/745 (19.9)	1.0	1.0
Luteal phase	136/526 (20.5)	1.04 (0.81-1.33)	1.04 (0.81-1.34)
**Parity:**			
0-1	55/277 (16.6)	1.0	1.0
2-4	237/943 (20.1)	1.27 (0.92-1.74)	1.16 (0.83-1.61)
5+	133/389 (25.5)	1.72 (1.21-2.44)	1.38 (0.94-2.01)
**Abortion:**			
No abortion	216/804 (21.2)	1.0	1.0
One abortion	96/338 (22.1)	1.05 (0.80-1.38)	0.99 (0.75-1.31)
2+ abortions	77/262 (22.7)	1.09 (0.81-1.47)	0.98 (0.72-1.33)
**Vaginal Douching:**			
Never/occasional	379/1457 (20.6)	1.0	1.0
Frequent	26/95 (21.5)	1.06 (0.67-1.66)	1.06 (0.67-1.67)

*Frequencies above and below the threshold for the top 20% quintile and percentage with high seroreactivity; **Odds ratios and respective 95% confidence intervals. First category in each variable is the referent; ***At the time of specimen collection: follicular (before 14 days) and luteal phase (after 14 days). Post-menopausal women and women with irregular cycles were not included in the analysis.

We also analyzed the associations between cervical HPV findings at enrollment and serological response to HPV16. Table 4 presents crude and age-adjusted ORs for positivity for cervical HPV DNA of different HPV types and examines the potential effect of single versus multiple infections, viral load, and infection by HPV types in HPV16-related (i.e., Alphapapillomavirus 9 species, which also includes HPV types 31, 33, 35, 52, 58, 67) as well as unrelated phylogenetic groupings (i.e., Alphapapillomavirus species 7, 3, 15). There was a strong association between being HPV16 DNA positive in the cervix and having high anti-HPV16 seroreactivity (age-adjusted OR = 3.86, 95%CI: 2.23-6.59). Partitioning this association between cases in which HPV16 was the sole type in the cervical sample and those in which it was found in coinfection with other types did not materially change the strength of the association (Table 4). There was no clear indication that the quantity of HPV16 DNA in the cervix was associated with seroreactivity; after controlling for age the strength of the association was as strong for women with less than one viral copy per cell as for those with more than 100 copies per cell. Serological response to HPV16 seemed to be mostly type-restricted since there was little to no evidence that infection with types other than HPV16 (except for phylogenetically-related Alphapapillomavirus 9 types) exerted any influence on HPV16 seroreactivity.

**Table 4:** **Associations between cervical HPV infection findings and serological reactivity for HPV16**

HPV infection variable	Categories	HPV16 seroreactivity 425/1624 (%)*	OR (95% CI)**	
			Unadjusted	Age-adjusted
HPV16 DNA	No	398/1593 (20.0)	1.0	1.0
	Yes	27/30 (47.4)	3.60 (2.11-6.13)	3.86 (2.23-6.59)
HPV16 DNA	Negative	398/1593 (20.0)	1.0	1.0
	Single infection	19/20 (48.7)	3.80 (2.01-7.19)	3.93 (2.07-7.48)
	Other types present	8/10 (44.4)	3.20 (1.25-8.17)	3.69 (1.44-9.47)
HPV16 viral load***	Negative	398/1593 (20.0)	1.0	1.0
	<1	8/11 (42.1)	2.91 (1.16-7.28)	3.10 (1.23-7.79)
	1-100	12/13 (48.0)	4.33 (1.96-9.58)	4.64 (2.09-10.3)
	100+	7/6 (53.9)	3.43 (1.14-10.3)	3.73 (1.24-11.2)
HPV18 DNA	No	423/1605 (20.9)	1.0	1.0
	Yes	2/18 (10.0)	0.42 (0.10-1.82)	0.45 (0.10-1.96)
HPV31 DNA	No	420/1604 (20.8)	1.0	1.0
	Yes	5/19 (20.8)	1.01 (0.37-2.71)	1.18 (0.43-3.21)
HPV33 DNA	No	423/1616 (20.8)	1.0	1.0
	Yes	2/7 (22.2)	1.09 (0.23-5.27)	1.13 (0.23-5.52)
HPV6/11 DNA	No	420/1606 (98.9)	1.0	1.0
	Yes	5/17 (1.1)	1.12 (0.41-3.06)	1.26 (0.46-3.44)
Non HPV16 Alpha PV 9****	No	400/1574 (20.3)	1.0	1.0
	Yes	25/50 (33.3)	2.00 (1.20-3.21)	2.17 (1.32-3.56)
Alpha PV 7	No	412/1566 (20.8)	1.0	1.0
	Yes	13/58 (18.3)	0.85 (0.46-1.57)	0.90 (0.49-1.67)
Alpha PV 3/15	No	416/1584 (20.8)	1.0	1.0
	Yes	9/40 (18.4)	0.86 (0.41-1.78)	0.87 (0.41-1.81)
Any HPV	No	338/1371 (19.8)	1.0	1.0
	Yes	87/252 (25.7)	1.40 (1.07-1.83)	1.52 (1.15-1.83)
Any HPV (non-HPV16)	No	338/1371 (19.8)	1.0	1.0
	Yes	60/222 (21.3)	1.10 (0.80-1.49)	1.19 (0.88-1.64)
Any HR-HPV	No	363/1466 (19.9)	1.0	1.0
	Yes	62/157 (28.3)	1.60 (1.16-2.19)	1.73 (1.25-2.39)
Any LR-HPV	No	400/1528 (20.8)	1.0	1.0
	Yes	25/95 (20.8)	1.00 (0.63-1.58)	1.06 (0.67-1.67)

*Frequencies above and below the threshold for the top 20% quintile and percentage with high seroreactivity; **Odds ratios and respective 95% confidence intervals. First category in each variable is the referent; ***Number of copies per cell; ****Alphapapillomavirus species 9 and other groupings: see text for details; viral load.

Finally, we attempted to identify the most parsimonious set of correlates of HPV16 seroreactivity via multivariate analyses. As shown in Table 5, model 1 includes all variables that were associated with seroreactivity in univariate or bivariate models. Model 2 is the minimum set of independent predictors after backwards elimination. Even when accounting for cervical HPV16 DNA positivity (as a binary variable, as indicators of mono and coinfections, or as viral load [data not shown]) seroreactivity increased monotonically with age (p value for trend <0.001) and with markers of increased sexual risk behaviors such as lifetime sexual partners, early age of first intercourse, and frequency of sex.

**Table 5:** Multivariate models for correlates of HPV16 seroreactivity*

Variable	Categories	Model 1	Model 2
Age (years)	<25	1.0	1.0
	25-34	1.36 (0.94-1.97)	1.52 (1.1-2.2)
	35-44	1.73 (1.15-2.61)	1.91 (1.28-2.87)
	45+	2.41 (1.48-3.93)	3.07 (1.86-5.06)
Income	1^st^ Quartile	1.0	
	2^nd^ Quartile	0.88 (0.63-1.23)	
	3^rd^ Quartile	0.81 (0.58-1.13)	
	4^th^ Quartile	0.78 (0.57-1.06)	
Smoking duration	Never	1.0	
	<10 years	0.71 (0.52-0.95)	
	> = 10 years	0.59 (0.44-0.79)	
Lifetime sexual partners	0-1	1.0	1.0
	2-3	1.76 (1.33-2.32)	1.44 (1.05-1.96)
	4-5	2.69 (1.89-3.84)	2.06 (1.39-3.05)
	6+	2.18 (1.40-3.4)	1.74 (1.09-2.79)
Age at first sexual intercourse	20-50	1.0	1.0
	18-19	1.50 (1.05-2.14)	1.46 (1.00-2.12)
	16-17	1.41 (0.98-2.03)	1.36 (0.94-1.96)
	<=15	1.64 (1.13-2.40)	1.71 (1.18-2.48)
Frequency of sex (weekly)	0-1	1.0	1.0
	2-3	1.07 (0.83-1.39)	1.05 (0.79-1.38)
	4-5	1.63 (1.07-2.47)	1.60 (1.05-2.45)
	6+	1.57 (0.96-2.66)	1.72 (1.01-2.94)
Condom Use	Never	1.0	
	Rarely	1.00 (0.73-1.35)	
	Sometimes/always	0.77 (0.57-1.02)	
Parity	0-1	1.0	
	2-4	1.20 (0.84-1.71)	
	5+	1.38 (0.86-1.95)	
HPV16 viral load	Negative	1.0	1.0
	<1	2.42 (0.88-6.76)	4.16 (1.35-12.78)
	1-100	4.19 (1.84-9.56)	4.03 (1.48-10.45)
	100+	3.73 (1.10-12.6)	3.18 (0.78-12.98)
HPV16 DNA co-infection	No HPV16	1.0	
	HPV16 alone	3.53 (1.76-7.10)	
	HPV16 + other type	3.30 (1.25-8.63)	

*Model 1: Mutually adjusted for all variables shown; model 2: most parsimonious set of independently explanatory variables via stepwise regression with backwards elimination (due to collinearity issues between HPV16 and viral load, only HPV16 viral load was retained as covariate for the models shown).

## Discussion

In this study we analyzed the epidemiologic correlates of strong anti-HPV16 seroreactivity at the enrollment visits of women from the Ludwig-McGill Cohort Study. We defined the serological reactivity as strong if the subject's average NAR was in the upper quintile of anti-HPV16 ELISA seroreactivity. This definition is probably more stringent than methods used in early reports, but associations with similar factors were identified. Our results revealed that strong HPV16 seroreactivity increased with age, and other factors that reflect HPV exposure, as number of lifetime sexual partners, younger age at first intercourse and higher frequency of sex.

The association of HPV16 serology with increasing number of sexual partners is well known [8],[22],[23]. We also observed a positive association between seroreactivity and lifetime number of sexual partners but not with number of recent sexual partners. This is in line with the fact that serology reflects HPV exposures that may have occurred cumulatively since the onset of sexual behavior, whereas current HPV DNA positivity reflects recent sexual exposure. Indeed, in previous analysis of this cohort, women with a greater number of sexual partners were more likely to have HPV infection, but likelihood of infection was more strongly influenced by the recent history of sexual activity [24]. Moreover, one cannot exclude the possibility that HPV infections in other anatomic sites could influence HPV seroreactivity, as previously described in a cohort study of HPV infection in men [25].

In our analysis, seroreactivity increased with age. Although this association was not consistently found in other investigations [23],[26], associations at specific age categories (e.g., 30-44 ys) were observed by other authors [8]. The association we observed with education and income, albeit non-significant upon adjustment, has not been observed in previous studies [23].

Although the Ludwig-McGill study is based on a multiple-measurement, prospective cohort investigation we focused on determinants of baseline seroreactivity using only serological results from a single time point (the enrollment visit), which prevented us from considering duration of infection in the analyses. This limitation in our ability to define when infections began prevented us from understanding the timing for serological responses to become observable following the initial exposure. Nevertheless, we observed a positive correlation between HPV16 seroreactivity and HPV16 DNA positivity alone or in coinfection with other types with strong evidence of type-restricted responses. We also observed some degree of cross-reactivity for infection with other types in Alphapapillomavirus 9 species (excluding HPV16), which can be expected because of the similarity of antigenic epitopes in L1 and L2 for members of that species.

Porras and colleagues (2010) reported that a high viral load for HPV16 is positively correlated with anti-HPV16 antibody titer, suggesting that seroconversion could be a consequence of an increase in antigen exposure [27]. We have not observed such a correlation; anti-HPV16 seroreactivity was uniformly high even for low HPV16 viral load levels. One possible explanation is that harboring a high viral load could reflect inability to control viral infection.

The relation between the use of contraceptives and HPV seropositivity has been controversial. In our study, neither use of contraceptives (oral, intra-uterine device or condom), nor parity were significant determinants of seroreactivity. A positive influence of hormonal contraceptives on HPV16 seropositivity was described before [23]. Specifically for HPV16, other studies described elevated risk of seropositivity with oral contraceptive use [22],[28]. We have found that current and prolonged smoking is associated with decreased HPV16 seroreactivity, and similar associations were already described [22],[26]. This could be a reflex of the immunosuppressive effect of smoking.

## Conclusion

On the basis of this cross-sectional analysis we observed that strong baseline seroreactivity was positively correlated with age, lifetime number of sexual partners, frequency of sex, and HPV16 viral load, and negatively associated with duration of smoking. Taken together our data suggest that HPV16 seroreactivity is determined by factors that reflect viral exposure.

## Authors' contributions

PSAS analyzed and interpreted the data and drafted the manuscript; AVR managed the databases performed the statistical analysis and contributed to the draft of the manuscript; JMGC and PT carried out the serology detection; AT performed the viral load measurements; ELF conceived the study design and data analysis strategy; LLV participated in the study design and supervised the study. All authors read and approved the final manuscript.

## Abbreviations

AvgNAR: Average NAR
CI: Confidence intervals
ELISA: Enzyme linked immuno sorbent assay
HPV: Human papillomavirus
HR HPV: High risk types of HPV
OD: Optical density
OR: Odds ratio
PBS: Phosphate buffered saline
PCR: Polymerase chain reaction
NAR: Normalized absorbance ratio
